# Chemically Defined Non-human Glycans Comprising Galactose-α1-3-Galactose (α-Gal) Epitopes Glycoengineered into the Fragment Antigen-Binding (Fab) Domain of Cetuximab Differentially Affect Human Anti-α-Gal Immunoglobulin E (IgE) Binding

**DOI:** 10.1021/acsptsci.5c00698

**Published:** 2026-03-18

**Authors:** Grayson Hatfield, Lioudmila Tepliakova, Roger Y. Tam

**Affiliations:** † Centre for Oncology, Radiopharmaceuticals and Research, Biologic and Radiopharmaceutical Drugs Directorate, 6348 Health Canada, Ottawa, Ontario K1A 0K9, Canada; ‡ Department of Chemistry, University of Ottawa, Ottawa, Ontario K1N 6N5, Canada

**Keywords:** Monoclonal antibodies, immunogenicity, glycosylation, non-human glycans

## Abstract

Glycosylation of monoclonal antibodies (mAbs) critically affects their effector function, stability, pharmacokinetics, and immunogenicity. Glycan structures can vary with cell type and culture conditions and are often considered key critical quality attributes that must be tightly controlled. While most mAbs are glycosylated only in the Fc-domain, cetuximab contains non-human glycans (galactose-α1-3-galactose, α-Gal) in its Fab-region, which can trigger hypersensitivity in patients with α-Gal-specific IgE. Previous studies linked bivalent α-Gal glycans to IgE binding, but the roles of other α-Gal glycans were unclear. Using glycoengineering, we herein incorporate α-Gal glycans found in commercial products and test their binding to patient-derived anti-α-Gal IgE. Unexpectedly, certain monovalent α-Gal glycans bound IgE as effectively as bivalent forms, and molecular modeling suggests that this may be attributed to interactions with the protein backbone. These findings provide important guidance for manufacturers and regulators in the development and evaluation of mAbs, biosimilars, and emerging glycoprotein therapeutics.

## Graphical abstract

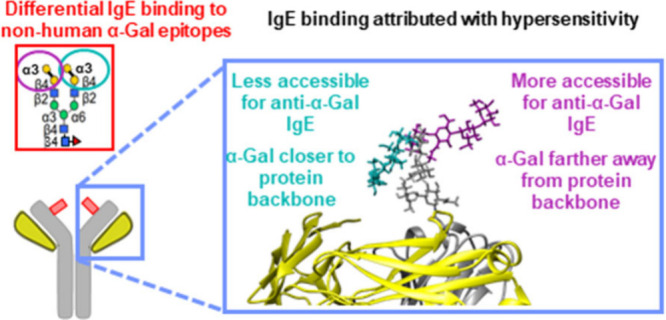

Antibody-based biotherapeutic drugs are used to treat various diseases, including cancer and anti-inflammatory conditions. Commercial manufacturing of these complex proteins typically uses non-human cells such as Chinese hamster ovary (CHO) or murine cells (SP2/0 or NS0) cells,^1,2^ and to a lesser extent other cells including *E. coli* ^3^ and yeast cells.^4^ Protein post-translational modification can alter protein bioactivity and stability, with glycosylation being among the most common. Glycosylation is controlled by dynamic interactions with glycosidases and glycosyltransferases encountered during translation in the endoplasmic reticulum and Golgi bodies, followed by those in the cytoplasm and extracellular microenvironment.^5^ Changes in cell culture conditions can alter glycan profiles,^6^ ultimately affecting its biological activities such as cell receptor binding.^7^ Therefore, glycoprofile analysis of monoclonal antibodies is often a critical quality attribute, and a thorough understanding of glycan properties is crucial to ensure product safety and potency.

Protein glycosylation by non-human cells can introduce non-human glycan structures including those comprising terminal galactosyl-α-1,3-galactosyl (referred herein as “α-Gal”) moieties via α-1,3-galactosyltransferase 1 (GGTA1).^8^ Recognizing that murine cells can incorporate α-Gal-containing glycans into the glycoprotein, antibody manufacturing has predominantly shifted to production in CHO cells, which lack GGTA1. However, incorporation of α-Gal glycans is still possible with CHO cells, which can express an ortholog enzyme to produce α-Gal structures.^9^ Their presence in cetuximab, a rare mAb therapeutic comprising glycosylation in the Fab domain, has been linked to hypersensitivity reactions in some patients who also have allergies to α-Gal-containing red meat.^10,11^ Previous studies have associated these allergic reactions with tick bites,^12^ which can cause sensitization to α-Gal glycans via increased production of anti-α-Gal IgE, the endogenous antibody that mediates allergic responses.^11^ Binding of host-generated anti-α-Gal IgE^11^ to α-Gal residues in the Fab domain of cetuximab is hypothesized to drive hypersensitivity.^13^ Using commercial cetuximab with heterogeneous glycans in both the Fab and Fc domains,^13^ and other commercial antibodies (*e.g*., infliximab, palivizumab) with low heterogeneous levels of Fc α-Gal glycans, it was hypothesized that IgE was unable to bind Fc α-Gal glycans. However, rare cases of anaphylaxis have been documented with infliximab,^14,15^ which has low levels of Fc α-Gal glycans^13^ but none in the Fab domain. To rationalize this discrepancy, we recently demonstrated that Fc-glycans with bivalent α-Gal structures can bind human anti-α-Gal IgE.^16^ With our ongoing interest in better defining the bioactivities of specific glycans to ensure antibody product safety, we set out to study how specific α-Gal glycan structures in the Fab domain of cetuximab contribute to human anti-α-Gal IgE binding.

Herein, we prepared a series of glycoengineered cetuximab analogues comprising homogeneous α-Gal-containing Fab glycans to study glycan structural properties in causing hypersensitivity via binding to human patient-derived anti-α-Gal IgE. These data are particularly useful for drug developers and regulatory evaluators in designing and evaluating drugs to limit the risk for hypersensitivity reactions. With the growing numbers of biosimilars that can be produced by manufacturers using different protocols compared to the innovator drug, as well as other antibody-based modalities using novel platforms, it is important to better understand which specific α-Gal structures may be most responsible for IgE-binding to ensure product safety.

We initially pursued synthesizing analogues comprising α-Gal glycans into the Fab domain with deglycosylated Fc domains, leaving a fucosylated GlcNAc residue bound to Asn-297 (Figure 1A, Table 1). Taking advantage of the transglycosylation activity of the EndoF3-D165A enyzme^17^ that can install glycans into the Fab domain of cetuximab, and the selectivity of EndoS glycosidases to cleave Fc-glycans, we first removed all glycans of both the Fab and Fc domains from commercial cetuximab (**1**) to produce deglycosylated cetuximab (**2**). To increase the reaction rate and simplify purification, we immobilized commercial EndoS and EndoF3, each produced as fusion proteins with chitin-binding domains (CBD), to chitin resin. Transglycosylation of both domains with α-Gal glycan oxazolines^16^ was performed (**3**), followed by cleavage of Fc-glycans with EndoS-CBD and chitin resin (**4**). As a control, an analogue comprising native Fab glycosylation with a deglycosylated Fc was also prepared (**5**).

**Figure 1:**
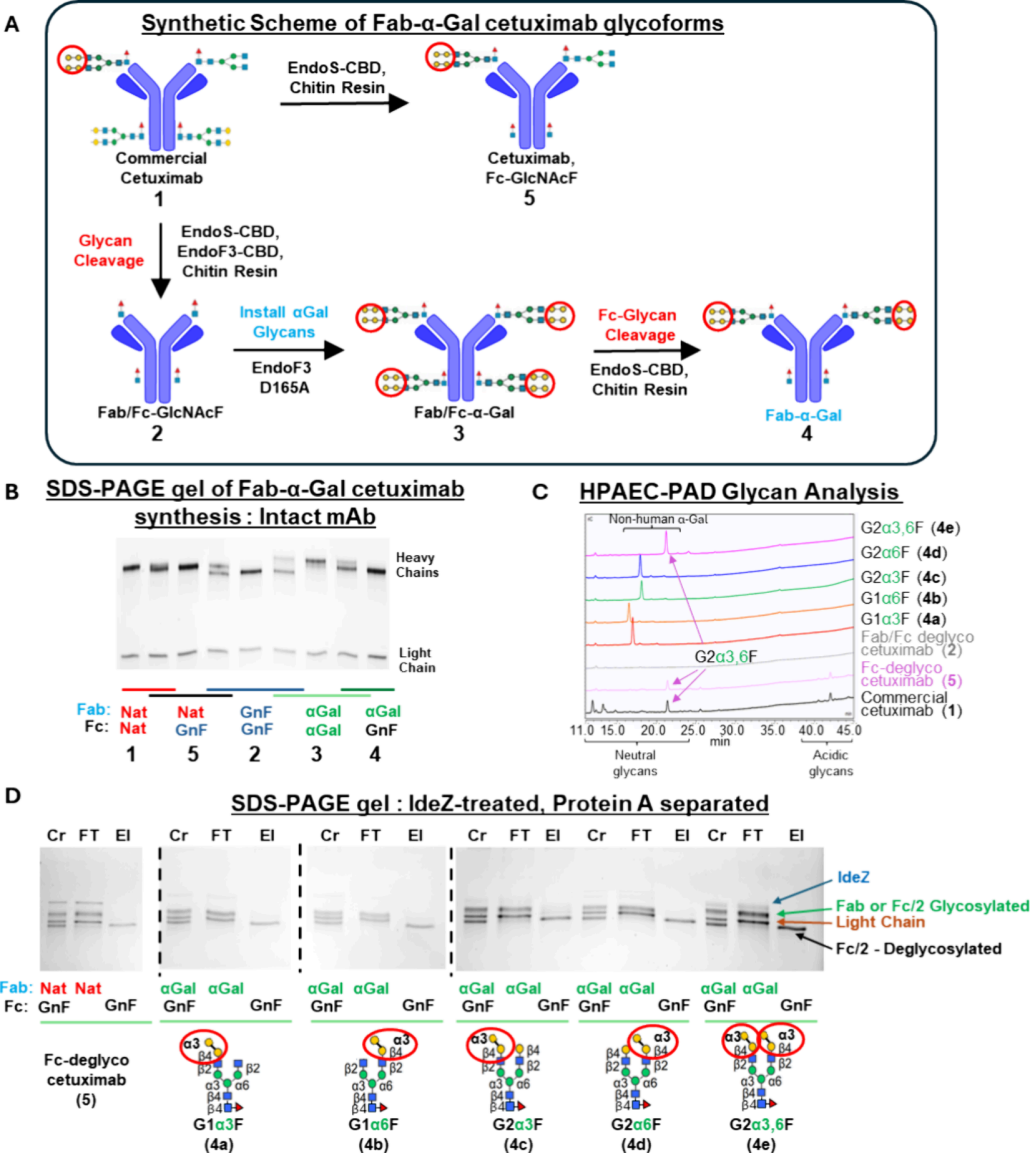
(A) Synthetic scheme of cetuximab with Fab α-Gal glycans and a deglycosylated Fc domain. Red circles highlight α-Gal epitopes. (B) SDS-PAGE gel showing commercial cetuximab starting material (**1**, lanes 1,2), deglycosylated cetuximab (**2**, lanes 4–6, GnF = fucosylated-GlcNAc), Fab/Fc-α-Gal intermediate (**3**, lanes 6–8), and Fab-α-Gal/Fc-deglycosylated cetuximab (**4**, lanes 8,9). Cetuximab with native (“Nat”) Fab glycosylation and Fc deglycosylation (**5**, lanes 2–4). (C) HPAEC-PAD glycan analysis of analogues following treatment with CTAB and PNGaseF from intact cetuximab, where α-Gal glycoforms (**4a**–**e**) show a dominant single peak. Deglycosylated cetuximab (**2**, gray trace) is absent of glycan peaks, while cetuximab with native Fab-glycosylation/Fc-deglycosylation (**5**, pink trace) shows bivalent α-Gal (G2­(α3,6)­F) and acidic glycans, consistent with literature.^13,16^ (D) SDS-PAGE gel showing site-specific glycosylation (Fab domain) and deglycosylation (Fc domain). Cleavage and separation of Fab and Fc domains was performed using IdeZ and Protein A resin, respectively. For each analogue, the crude reaction (Cr) comprises IdeZ, glycosylated Fab, light chain, and deglycosylated Fc domains. Separation by protein A results in the latter in elution (El) fractions, while the remaining unbound components are present in the flow-through (FT).

**Table 1:**
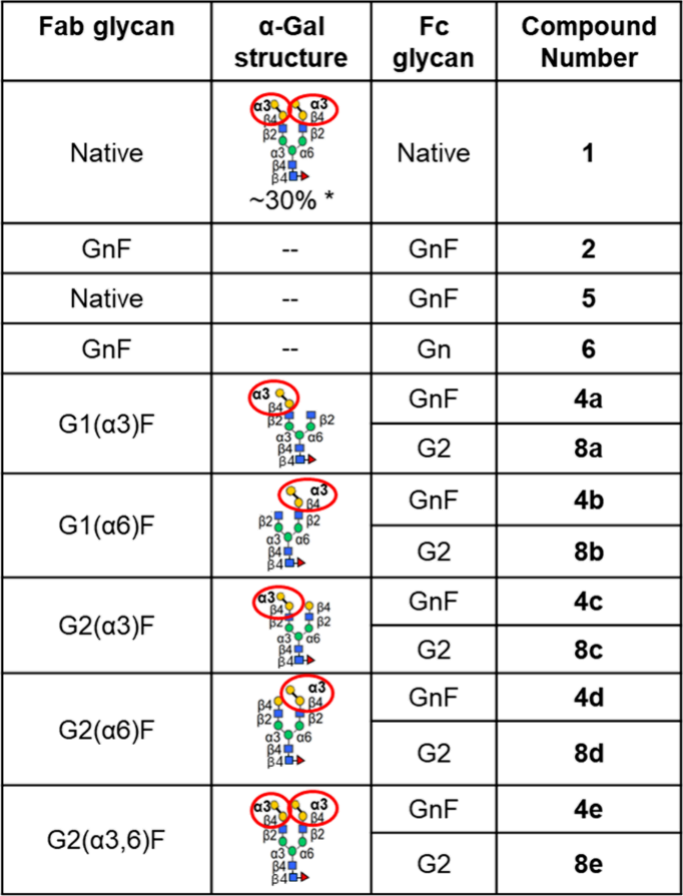
Various Cetuximab Glycoforms Prepared and Tested for Binding to EGF-R and Anti-α-Gal IgE Are Shown with Their Corresponding Compound Numbers[Table-fn tbl1-fn1]

* The percentage of bivalent α-Gal glycans is relative to other glycans in the Fab domain, and is consistent with literature values.^16^

Figure 1B shows band migration shifts using reducing SDS-PAGE at each glycan remodeling step. To ensure that shifts were due to molecular weight changes and not fluctuations in gel migration within the gel, multiple samples were loaded into the same lane to serve as an internal reference migration standard. Transglycosylated glycans were characterized by performing deglycosylation with PNGaseF in the presence of cetyl triammonium bromide (CTAB), which is required to efficiently cleave both Fab and Fc glycans.^13^ Glycan analysis (Figure 1C) by high pH anionic exchange chromatography-pulsed amperometric detection (HPAEC-PAD) shows that native commercial cetuximab (**1**) is expectedly heterogeneous with multiple glycan peaks, while Fc-deglycosylated cetuximab (**5**) comprises mostly bivalent α-Gal glycan (G2α3,6F, with one α-Gal epitope in each of its α3 or α6 antenna) and acidic glycans at 21 and 42 min, respectively, both of which are known to predominate in the Fab domain.^13,16^ In contrast, Fab-glycoengineered α-glycans (**4a**–**e**, Table 1) present as a single peak, consistent with our previous report of glycoengineering these glycans into the Fc domain.^16^ To confirm that α-Gal glycosylation occurs only in the Fab domain after the final Fc deglycosylation step with EndoS-CBD, final compounds (**4a**–**e**, **5**) were treated with IdeZ, a protease that selectively cleaves between Fab and Fc domains, and separated these domains using Protein A affinity chromatography, which specifically binds to Fc domains. Figure 1D shows that the crude reaction mixture comprises IdeZ, Fab, and Fc heavy chains that are of similar molecular weight when both are glycosylated and a light chain. Deglycosylation of either heavy chain fragment lowers the band to a position below the light chain. Analysis of fractions unbound by Protein A (*i.e*., ‘flow through’, FT) consistently shows IdeZ, the Fab heavy chain, and the light chain. Importantly, a band lower than the light chain (which would indicate Fab deglycosylation) is not observed. In the fraction bound by Protein A (*i.e*., ‘elution’, El), only a single band below the light chain is observed, indicating that the Fc domain is completely deglycosylated.

Next, we prepared a series of Fab α-Gal glycans with Fc domains glycosylated with afucosylated glycans that have terminal galactose residues (G2), as shown in Figure 2A. This strategy involved first cleaving glycans and core-fucose residues of the Fc domain with EndoS-CBD and AlfC fucosidase, respectively, while the Fab domain remained glycosylated. Selectivity is achieved as defucosylation occurs faster with deglycosylated vs. glycosylated glycans. Following the removal of these enzymes, EndoF3-CBD addition deglycosylated the Fab domain while maintaining the core-fucose residue (**6**). Reaction with α-Gal glycan oxazolines and EndoF3-D165A selectively transglycosylates the Fab domain (**7**) and not the Fc domain, which lacks the fucose required for transglycosylation. The human-type biantennary bivalent β1,4-galactose glycan (G2) was then transglycosylated into the Fc domain using EndoS2-D184 M to produce glycoforms (**8a**–**e**).^18^

**Figure 2:**
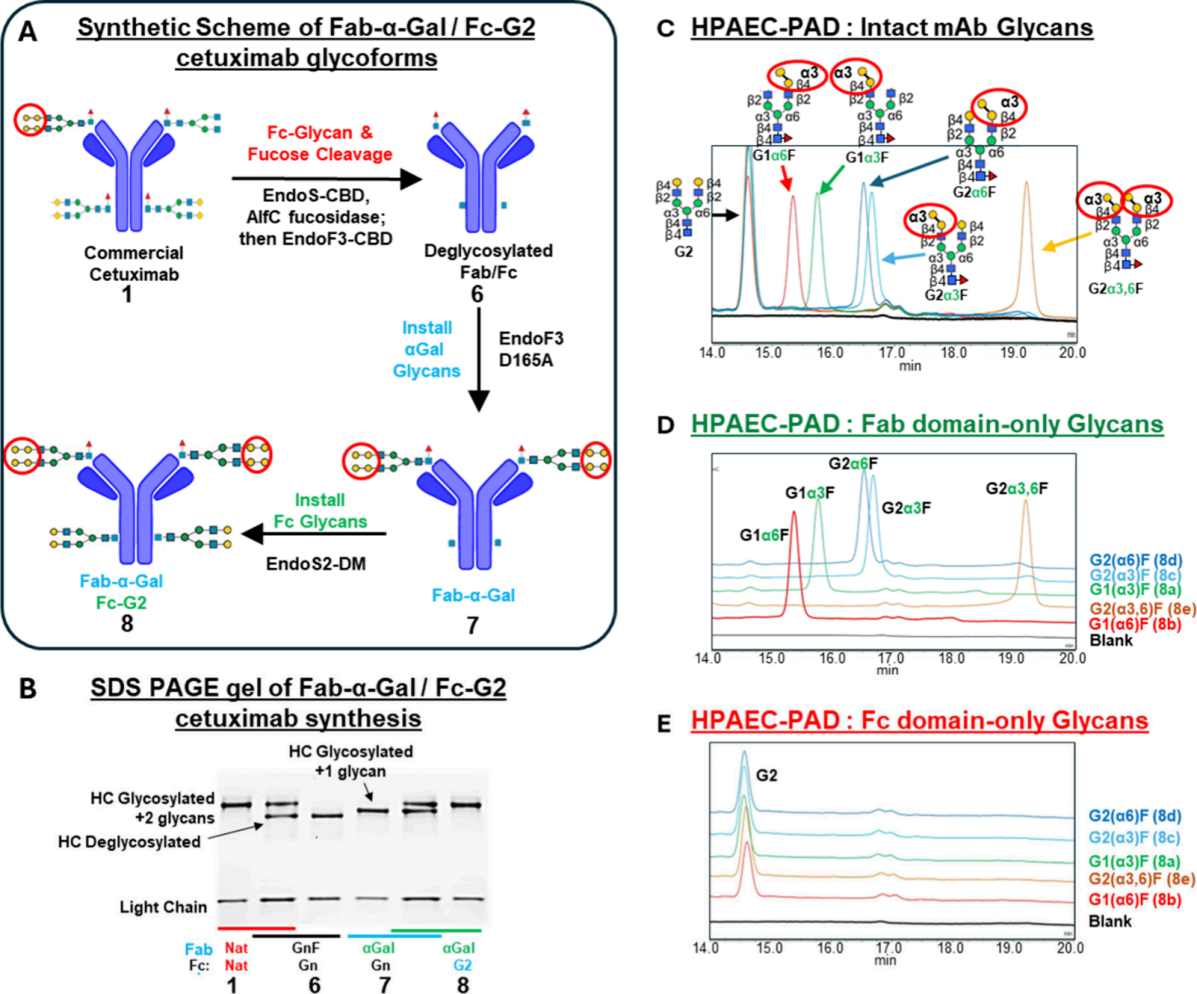
(A) Synthetic scheme of cetuximab with Fab α-Gal glycans and afucosylated, glycosylated Fc domain. (B) SDS-PAGE gel showing starting material cetuximab (**1**, lanes 1,2), afucosylated/deglycosylated cetuximab (**6**, lanes 2,3), Fab α-Gal glycosylated intermediate (**7**, lanes 4,5), and Fab-α-Gal/Fc-afucosylated/glycosylated cetuximab (**8**, lanes 5,6). (C–E) HPAEC-PAD glycan analysis of each cetuximab analogue following treatment with CTAB and PNGaseF from (C) intact cetuximab, showing the presence of both Fc-G2 and Fab-α-Gal glycans, and IdeZ-treated and protein-A separated (D) Fab and (E) Fc domains.

SDS-PAGE analysis at each synthetic step shows that the respective changes in band migration occur as expected (Figure 2B). HPAEC-PAD analysis of the intact final products (**8a**–**e**) show comparable ratios between Fc-G2 and the various α-Gal glycans in the Fab domains (Figure 2C). To confirm glycan specificity in each domain, samples were treated with IdeZ and separated by Protein A chromatography, and each domain was analyzed for their glycan content by HPAEC-PAD. Figure 2D,E shows that each Fab and Fc domain comprises exclusively α-Gal or G2 glycans, respectively.

To ensure that the transglycosylation processes did not affect the targeting bioactivity of glycoengineered cetuximab, its binding affinity to its target antigen, EGF-R, was determined by ELISA. As controls, commercial cetuximab (**1**) was compared to analogues deglycosylated at the Fc-only (**5**) or both Fab- and Fc-domains (**2**) (Figure 3A). No significant difference was observed, indicating that the glycosylation state of the Fc- or the Fab-domains did not significantly affect its binding to EGF-R, which is known to be driven by protein–protein interactions of the heavy and light chains of cetuximab (and away from the Fab glycosylation site Asn-88) with domain III of EGF-R.^19^ Next, assessing the binding of all Fab-α-Gal cetuximab samples (**4a**–**e**, **8a**–**e**) and the Fc-only deglycosylated cetuximab (**5**) control also showed no significant differences compared to each other (Figure 3B,C), indicating that the enzymatic transglycosylation processes did not negatively affect the overall protein conformation, and is consistent with previous similar glycoengineering strategies that did not significantly affect EGF-R binding.^17^

**Figure 3:**
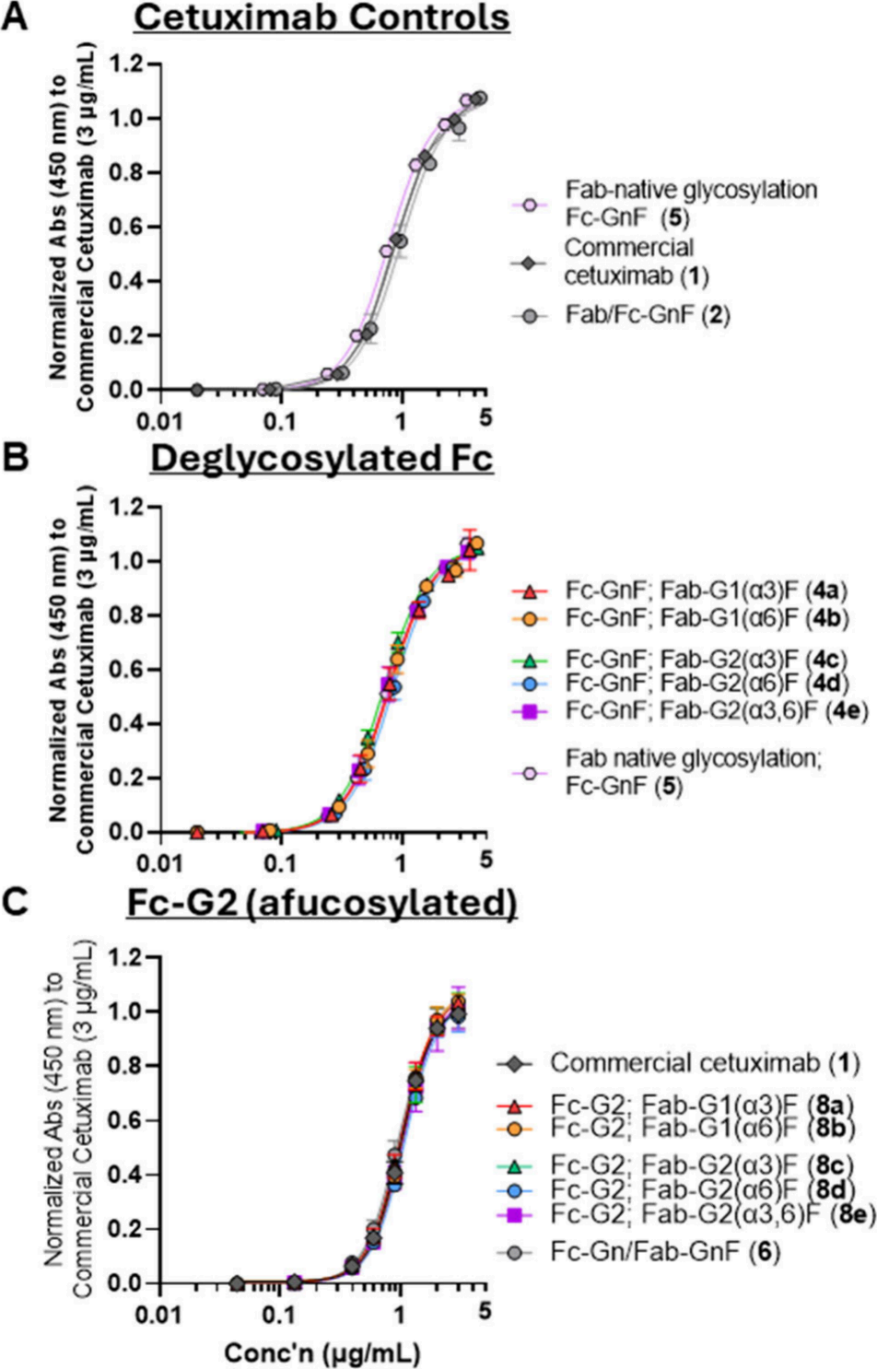
Binding of cetuximab glycoforms with EGF-R by ELISA for (A) cetuximab controls (**1**,**2**,**5**), and remodelled cetuximab with Fc domains that are (B) deglycosylated (**4a**–**e**), and (C) afucosylated/glycosylated with G2 (**8a**–**e**). No significant differences in binding are observed, indicating that neither the glycosylation profiles nor the transglycosylation reactions affect target binding.

Next, we determined the immunogenicity of different Fab α-Gal glycans using an anti-α-Gal IgE (16D9) monoclonal antibody derived from a human patient who had hypersensitivity to red meats^16^ in an ELISA binding assay, which has precedent to reflect clinically relevant IgE interactions.^20^ This human IgE is produced using human hybridoma technology,^21^ and although the role of IgE glycosylation in cetuximab binding is unclear and may vary between patients, this anti-α-Gal IgE (16D9) has high specificity for the α-Gal epitope (as determined by an ImmunoCAP assay with natural Gal-α1-3-Gal glycans).

Examining the effect of Fc glycosylation on IgE binding, we observed that commercial cetuximab (with a mixture of fucosylated Fc glycans (**1**) dark gray diamonds) exhibited slightly lower binding compared to those with deglycosylated Fc domains (**5**, pink) at high concentrations (Figure 4A). This is notably in contrast to EGF-R binding, which was not affected by Fc (or Fab) glycosylation states (Figure 3A). This difference may be attributed to increased exposure of the Fab-domain (and therefore glycosylation site) in the Fc-deglycosylated form, which has been reported to be more flexible compared to Fc-glycosylated mAbs.^22^ The significance of steric hindrance of the Fab-glycosylation site (Asn-88) is also evident by the need for additional denaturing conditions (*i.e*., CTAB and heat) in enzymatic deglycosylation reactions with PNGase F to proceed efficiently.^13^ Together, this suggests that the increased flexibility of the Fab and Fc domains and steric hindrance around the Fab α-Gal glycans affect α-Gal IgE binding.

**Figure 4:**
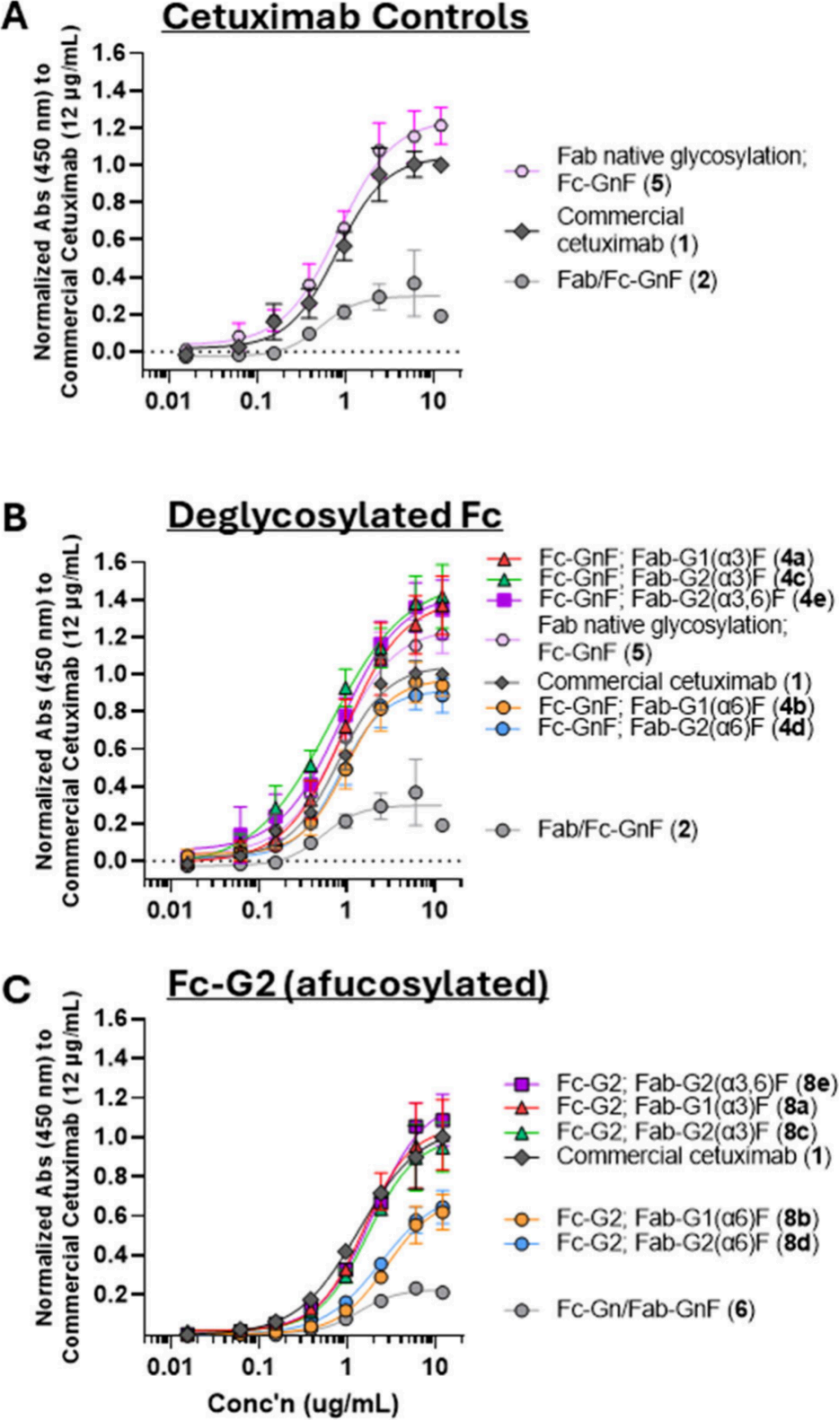
Binding of cetuximab glycoforms with anti-α-Gal IgE (16D9) by ELISA, for (A) cetuximab controls (**1**, **2**, **5**), remodeled cetuximab with Fc domains that are (B) deglycosylated (**4a**–**e**), and (C) afucosylated/glycosylated with G2 (**8a**–**e**). In controls (A), cetuximab with deglycosylated Fc (with native Fab glycoprofiles, **5**) showed higher binding than that of commercial cetuximab (with native Fc and Fab glycoprofiles). For all Fc-glycoprofiles (**B**, C) with Fab-α-Gal glycans, glycans with α-Gal glycans in the α3 antenna (*ex*. Fab-G1­(α3)­F (red, **4a**, **8a**)/G2­(α3)F (green, **4c**, **8c**)/G2­(α3,6)F (magenta, **4e**, **8e**)) consistently exhibit higher binding than those without (*ex*. Fab-G1­(α6)F (orange, **4b**, **8b**)/G2­(α6)F (blue, **4d**, **8d**)).

Surprisingly, α-Gal glycan epitopes in the α3 antenna were the predominant factor in binding to human derived anti-α-Gal IgE antibodies, and the α6 antenna contributed to less binding, albeit still higher than the negative deglycosylated control (Figure 4B,C). This pattern is observed for all analogues, regardless of the Fc glycosylation profile (*i.e*., deglycosylated (Figure 4B) vs. afucosylated (G2, Figure 4C); presence of the β1–4 galactosyl residue on the opposite α6 antenna (*i.e*., G1­(α3)F vs G2­(α3)­F, red vs green triangles) did not significantly affect IgE binding for any Fc series of analogues. Our data showing that the α-Gal glycans in the α6 has less binding than those in the α3 antenna may be due to different interactions of each with the protein backbone, consistent with a recent report^23^ showing that terminal α-Gal residues on either antenna interacted differently with the protein backbone of cetuximab.

To gain insight into how subtle changes in Fab α-Gal structures cause the observed changes in anti-α-Gal IgE binding, we used the GlycoShape and Re-Glyco molecular modeling software tools^24^ to model the bivalent α-Gal glycan (G2­(α3,6)­F, ID G38882R) at the native glycosylation site (Asn-88) of the Fab heavy chain of cetuximab (PDB ID 1YY8 ^19^). By plotting the anomeric center (C1) of the terminal α-Gal residues of the α3- or α6-antenna (magenta or cyan, respectively) for 500 glycan conformations, differences in orientations relative to the protein backbone are observed (Figure 5A,B). A representative snapshot of a glycan conformation is shown in Figure 5C,D. The distance between each α-Gal C1 with the nearest Cα of the protein backbone is shown in Figure 5E, and together these data clearly show that the α-Gal residues of the α6 antenna are closer to the protein backbone compared to those in the α3-antenna. Relating this to our observed anti-α-Gal IgE binding data, this suggests that protein-glycan interactions between anti-α-Gal IgE and α-Gal residues are sensitive to steric hindrance around the α-Gal residues.

**Figure 5:**
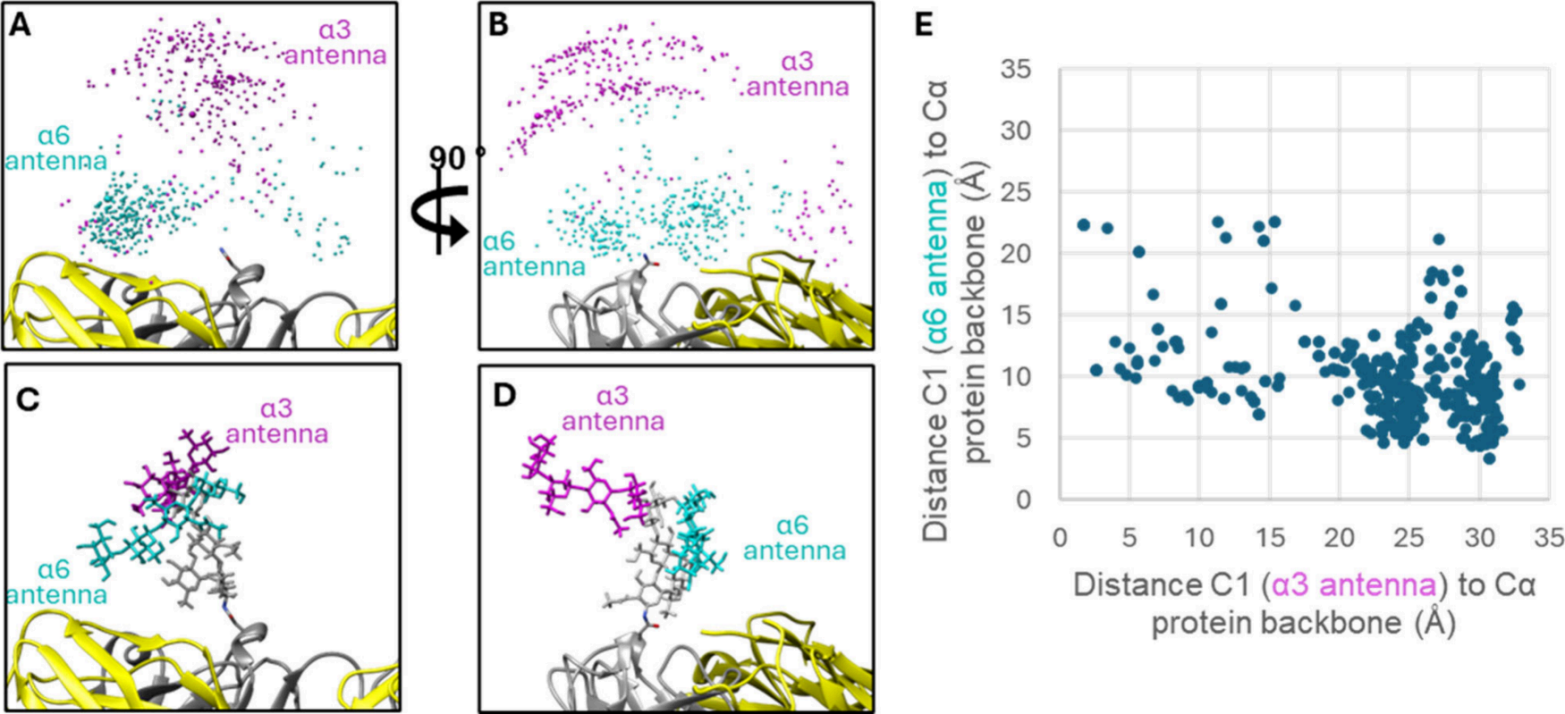
GlycoShape rendering of Fab glycan G2­(a3,6)F (ID G39992DR), onto Asn 88 of the Fab heavy chain (white, PDB ID 1YY8 ^24^). Yellow = Fab light chain. (A, B) Point cloud of the anomeric C1 of each terminal α-Gal residue of the α3 antenna (magenta) and the α6 antenna (cyan). (C, D) Representative snapshot of the glycan conformations. (E) The measured distances between C1 of each antenna with the Cα of the nearest amino acid residue.

By using glycoengineering strategies to prepare more homogeneous glycoforms of the monoclonal antibody cetuximab, we herein provide detailed structure–function relationships between α-Gal-containing glycans in the Fab domain with their potential for causing hypersensitivity via binding to human patient-derived anti-α-Gal IgE antibodies. We demonstrate this is predominantly due to a common structural characteristic among biantennary Fab glycans, namely, its presence in the α3 antenna, and that glycan-protein interactions with the Fab protein backbone may sterically hinder IgE from the α6 antenna. Surprisingly, these relative binding affinities of α-Gal epitopes to IgE differ from their presence in the Fc domain,^16^ where bivalent G2­(α3,6)F glycans exhibited greater binding compared to monovalent α-Gal glycans. These latter results are consistent with a previous report^13^ that bivalent α-galactosylated glycans from cetuximab digested with proteinase K had higher IgE affinity than mono-α-galactosylated glycans generated from proteinase K-digested infliximab or a linear a α-Gal trisaccharide (Gal-α1,3-Gal-β1,4-GlcNAc). An explanation may be that proteinase K digestion abolished any glycan-protein interactions and exposes both α-Gal epitopes for greater exposure to IgE. Together, these data suggest the importance of glycan-protein interactions in IgE binding, and future work to determine how this applies to different biotherapeutic glycoproteins is needed. Moreover, additional novel approach methodology studies are warranted to further validate these findings and study downstream hypersensitivity mechanisms.

These results are important for the development and regulatory evaluation of biosimilars and antibody-based modalities using novel platforms, as well as conventional CHO cells, which have been shown in rare cases to be able to incorporate non-human α-Gal glycan epitopes.^9^ As protein glycosylation is sensitive to manufacturing conditions, thorough glycan characterization is important to ensure product quality and limit the risk of adverse immunogenic effects. Our data highlights the need to distinguish human vs. non-human glycan isomers (*e.g*., G2F vs G1­(α3)­F, respectively) which have the same molecular weights but can differ in immunogenic activities. Although challenging, using complementary orthogonal analytical assays such as enzymatic glycan mapping (*i.e*., with α1-3,4,6 galactosidase) and LC/MS and HPAEC-PAD analyses can improve glycan structural elucidation. Incorporating such strategies support safer, more consistent biologics and facilitate regulatory confidence in the quality and safety of antibody-based products.

## Materials and Methods

### Site-Specific Transglycosylation of Cetuximab

#### Deglycosylated Fc Analogues (**4a**–**e**)

Cetuximab (Erbitux, Imclone LLC/Eli Lilly, DIN#02271249) was purchased from McKesson Canada. Chitin resin (50 μL slurry, New England Biolabs, catalog no. S6651S) was washed twice with 50 mM Tris buffer (pH 7.3), and EndoS-CBD (1.0 μL, New England Biolabs, catalog no. P0741S) and EndoF3-CBD (1.0 μL, New England Biolabs, catalog no. P0771S) were added. Cetuximab (**1**, 1.0 mg in 500 μL) was added and incubated (37 °C, overnight) to deglycosylate Fc and Fab domains. Reaction progress was monitored by reducing SDS PAGE. Following completion, the reaction mixture was passed through a solid phase extraction (SPE) tube to remove chitin-bound enzymes. The remaining deglycosylated cetuximab (**2**) was diafiltered (30 kDa MWCO Amicon tube, Tris buffer) to 18 mg/mL.

To 200 μg of deglycosylated cetuximab (**2**, 11 μL), EndoF3-D165A (2.0 μL, 3 mg/mL, GlycoT Therapeutics, Cat#GT005) was added. Twenty-seven nmol (5 eq relative to glycosylation sites) of lyophilized α-Gal glycan oxazoline, prepared as previously described,^16^ was resuspended in 0.5 μL of Tris buffer and added to the reaction, followed by incubation (30 °C, 45 min). Another 107 nmol of α-Gal oxazoline in 1.5 μL Tris buffer was added and reaction continued for at least another 2.25 h until reaction completion, followed by purification Protein A chromatography.^16^ The Fc was selectively deglycosylated using EndoS-CBD (0.5 μL) bound to 50 μL of chitin resin (37 °C, overnight) and purified by SPE and Protein A chromatography.

#### Afucosylated, Glycosylated Fc (G2) Analogues (**8a**–**e**)

Using a similar workflow, 3.0 mg commercial cetuximab was deglycosylated and defucosylated using EndoS2-D184M^25,26^ (30 μg) and α-Fucosidase 29A (*Lc*Fuc29A) (100 μg, NZyTec Cat#CZ05664) (37 °C, 20 h). The Fc-deglycosylated intermediate was purified by Protein A chromatography and diafiltered (50 mM sodium acetate, pH 4.5) to 4.0 mg/mL. The Fab domain was deglycosylated with EndoF3-CBD (2.0 μL, 25 °C, 20 h), followed by addition of chitin resin slurry (100 μL, 4 °C, 30 min) and SPE purification. The flow-through was diafiltered (Tris buffer) to 11 mg/mL.

Fab transglycosylation using EndoF3-D165A was performed as above, purified by Protein A chromatography, buffer exchanged into Tris buffer, and concentrated to 2 mg/mL. Fc transglycosylation was performed by adding EndoS2-D184 M (1.5 μg) to Fab-αGal-glycosylated mAb (150 μg) and incubated at 25 °C. A 60 nmol (30 eq relative to available glycosylation sites) aliquot of G2 glycan oxazoline^26^ was resuspended in Tris buffer (2.0 μL), and added in four equal portions every 30 min. Completed reactions were purified by Protein A chromatography.

### Molecular Modeling

To model the relative conformations of α-Gal epitopes within the Fab heavy chain of cetuximab, we chose the bivalent glycan G2­(α3,6)F glycan (with α-Gal epitopes on each antenna, ID G39992DR) within the GlycoShape Glycan Database, and incorporated this into Fab glycosylation site (Asn-88 of PDB ID 1YY8)^19^ using the Re-Glyco tool.^24^ An ensemble size of 500 conformations and an Effort Level of 20 were used. Resulting PDB files were then analyzed using the UCSF ChimeraX software.^27^

## Supplementary Material



## Notes

Methods and Materials for HPAEC-PAD analysis and ELISA assays, and Supplemental Figures S1, S2 showing SDS-PAGE gels for antibody glycoform intermediates **3a**–**e** and final products **4a**–**e** and **8a**–**e** (PDF)

## Abbreviations

α-Gal: galactosyl-α1-3-galactose
mAb: monoclonal antibody
EGF-R: epidermal growth factor receptor
