# Supplementary material for: Chemically Defined Non-human Glycans Comprising Galactose-α1-3-Galactose (α-Gal) Epitopes Glycoengineered into the Fragment Antigen-Binding (Fab) Domain of Cetuximab Differentially Affect Human Anti-α-Gal Immunoglobulin E (IgE) Binding
Source: ACS Pharmacol Transl Sci. 2026 Mar 18;9(4):880–7. doi: 10.1021/acsptsci.5c00698 (PMC13077488; doi:10.1021/acsptsci.5c00698)
Supplement: Supplementary file 1 [file pt5c00698_si_001.pdf]

Supporting Information

Chemically-defined non-human glycans comprising galactose-α1-3-galactose (α-Gal) epitopes glycoengineered into the fragment antigen-binding (Fab) domain of cetuximab differentially affect human anti-α-Gal immunoglobulin E (IgE) binding

Grayson Hatfield,[1] Lioudmila Tepliakova,[1] Roger Y. Tam[1,2]*

[1] Centre for Oncology, Radiopharmaceuticals and Research, Biologic and Radiopharmaceutical Drugs Directorate, Health Canada, Ottawa, Ontario K1A 0K9, Canada

[2] Department of Chemistry, University of Ottawa, Ontario K1N 6N5,Canada

** corresponding author e-mail: Roger.tam@hc-sc.gc.ca*

Table of Contents

1. **Materials and Methods………………………………………….…………………… S3**
   - i. **Glycan Characterization by High pH Anion-Exchange Chromatography - Pulsed Amperometric Detection (HPAEC-PAD)……..… S3**
   - ii. **Binding affinity assays by ELISA……………………………………….….……..S3**
2. **Supplemental Figure 1 ……………………………………………………….…..…S5**
3. **Supplemental Figure 2…………………………………………………….….…..…S5**
4. **Reference……………………….……………………………………….…………..…S5**

Materials and Methods:

Glycan Characterization by High pH Anion-Exchange Chromatography - Pulsed Amperometric Detection (HPAEC-PAD)

Deglycosylation of intact or Fab/Fc domains of cetuximab was performed as previously described using cetyl trimethylammonium bromide (CTAB, TCI America, Cat# H0081) and PNGaseF (New England Biolabs, Cat # P0705).[1] Fab and Fc domains were cleaved and separated using IdeZ (New England Biolabs, Cat# P0770S) and Protein A resin chromatography, respectively. Glycans were purified by activated carbon and then analyzed by HPAEC-PAD using an ICS- 6000 HPAEC PAD instrument (Dionex) as previously described.[1] In brief, a Dionex CarboPac PA200 IC column (3 mm × 250 mm, 5.5 µm particle size, Cat # 062896), a Gold Standard PAD waveform with an AgCl electrode, and mobile phases comprising 200 mM NaOH (MP-A), 150 mM NaOAc in 200 mM NaOH (MP-B) and MQ H_2O (MP-C) were used. Glycan separation was performed using the following gradient: 40/0/60 % to 35/10/55 % (MP-A/MP-B/MP-C) over 22.0 min, then gradual increases to 35/15/50 % (4 min), 35/20/45% (4 min), 30/30/40 % (8 min), 27.2/47/25.8% (8 min). The column was regenerated using flushes with 0/100/0 % (1 min), followed by re-equilibration to 40/0/60 % (10 min).

Binding affinity assays by ELISA

Binding experiments by ELISA were performed as previously described.[1] In brief, serial dilutions of cetuximab samples were prepared in 100 mM bicarbonate buffer pH 9.6 and immobilized onto MaxiSorp plates (ThermoFisher, Cat# 439454) overnight at 4°C, at 50 µL/well. In general, samples were washed with PBS containing 0.05% Tween (PBS-T, pH 7.4), followed by addition of the blocking agent (1 h), and then replaced with 50 µL of the primary antibody for

2 h at room temperature. Following washing with PBS-T (pH 7.4), 50 µL of the secondary antibody was added for 1 h. Wells were then washed with PBS-T (pH 7.4) and 50 µL TMB substrate (Cell Signal Technologies, Cat# 7004P6) was immediately added. The reaction was stopped by adding 50 µL of 2 M H_2SO_4 and immediately imaged on a plate reader (450 nm). For each assay, three separate replicates were performed on different days.

For EGF-R binding, recombinant human EGFR-His-tag protein (R&D Systems, Cat# 11302-ER-050) was used as the primary antibody at a 1/5,000 dilution, with 1% bovine serum albumin (BSA, Sigma, Cat # A3059-100 G) in PBS-T (pH 7.4) as the blocking agent and antibody dilution buffer. HRP-conjugated rabbit anti-His-Tag antibody (ABclonal, Cat# AE104-50UL) was used as the secondary antibody, at a 1/20,000 dilution.

For anti-α-Gal IgE binding, anti-α-Gal IgE 16D9 (InBio, Cat#E-16D9) was used as the primary antibody at a 1/1,000 dilution and 1 % human serum albumin (HSA, Millipore Sigma, Cat# 12666) in PBS-T (pH 7.4) as the blocking agent and antibody dilution buffer. HRP-conjugated goat anti-human IgE antibody (Invitrogen, Cat# PISA510261) was used as the secondary antibody at a 1/3,000 dilution.

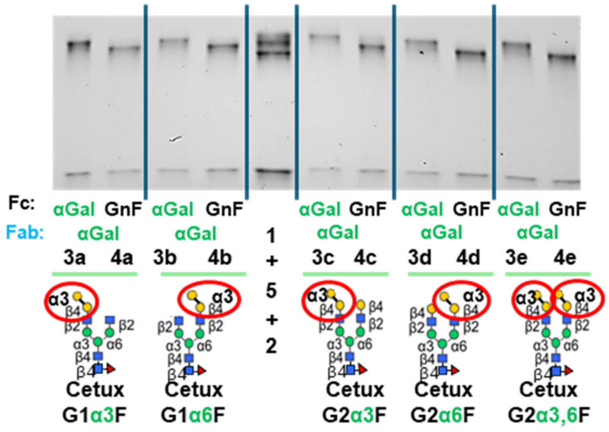

Supplementary Figure S1. Reducing SDS-PAGE gel image of each Fab-α-Gal cetuximab glycoform as the Fc-glycosylated intermediate (**3**) and Fc-deglycosylated (GnF) product (**4**). As a reference, lane 5 is co-spotted with native cetuximab (**1**), and deglycosylated cetuximab controls (**2**, **5**) to show the relative migration of all three bands together in one lane.

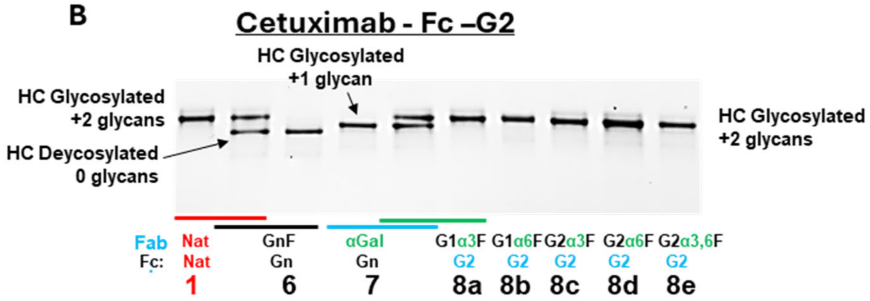

Supplementary Figure S2. Reducing SDS-PAGE gel image of each Fab-α-Gal cetuximab glycoform with an Fc containing the afucosylated G2 glycan (**8a-e**). The relative band migration is consistent with native cetuximab (**1**) which has the same number of glycans.

References

(1) Hatfield, G.; Tepliakova, L.; Tran, J.; Lu, H.; Gilbert, M.; Tam, R. Y. Bivalent non-human gal-alpha1-3-gal glycan epitopes in the Fc region of a monoclonal antibody model can be recognized by anti-Gal-alpha1-3-Gal IgE antibodies. *MAbs* **2023**, *15* (1), 2239405. DOI: 10.1080/19420862.2023.2239405
